# Mediterranean Diet and Obesity-related Disorders: What is the Evidence?

**DOI:** 10.1007/s13679-022-00481-1

**Published:** 2022-09-30

**Authors:** Giovanna Muscogiuri, Ludovica Verde, Cem Sulu, Niki Katsiki, Maria Hassapidou, Evelyn Frias-Toral, Gabriela Cucalón, Agnieszka Pazderska, Volkan Demirhan Yumuk, Annamaria Colao, Luigi Barrea

**Affiliations:** 1 Dipartimento di Medicina Clinica e Chirurgia, Endocrinology Unit, University Federico II, Naples, Italy; 2 Centro Italiano per la cura e il Benessere del paziente con Obesità (C.I.B.O), Department of Clinical Medicine and Surgery, Endocrinology Unit, University Medical School of Naples, Naples, Italy; 3 Cattedra Unesco “Educazione alla salute e allo sviluppo sostenibile”, University Federico II, Naples, Italy; 4 Division of Endocrinology, Metabolism and Diabetes, Istanbul University-Cerrahpaşa, Cerrahpaşa Medical Faculty, Istanbul, Turkey; 5 Department of Nutritional Sciences and Dietetics, International Hellenic University, Thessaloniki, Greece; 6 School of Medicine, Universidad Católica Santiago de Guayaquil, Av. Pdte. Carlos Julio Arosemena Tola, Guayaquil, 090615 Ecuador; 7 Escuela Superior Politécnica del Litoral, ESPOL, Lifescience Faculty, ESPOL Polytechnic University, Campus Gustavo Galindo Km. 30.5 Vía Perimetral, P.O. Box 09-01-5863, Guayaquil, Ecuador; 8 Division of Endocrinology, Metabolism, and Diabetes-Department of Internal Medicine, Cerrahpasa Medical School, Istanbul University-Cerrahpasa, Istanbul, 34098 Turkey; 9 Dipartimento di Scienze Umanistiche, Università Telematica Pegaso, Naples, 80143 Italy

**Keywords:** Mediterranean diet, Obesity, Obesity-related disorders, Cardiovascular diseases, Type 2 diabetes, Dyslipidemia

## Abstract

***Purpose of Review*:**

Obesity is a chronic disease, a major public health problem due to its association with non-communicable diseases and all-cause mortality. Indeed, people with obesity are at increased risk for a variety of obesity-related disorders including hypertension, dyslipidemia, type 2 diabetes mellitus, cardiovascular disease, and several cancers. Many popular diets with very different macronutrient composition, including the Mediterranean diet (MD), have been used, proposed, and studied for prevention and management of obesity. In particular, MD has been the subject of countless studies over the years and now boasts a large body of scientific literature. In this review, we aimed to update current knowledge by summarizing the most recent evidence on the effect of MD on obesity and obesity-related disorders.

***Recent Findings*:**

The negative effects of obesity are partly reversed by substantial weight loss that can be achieved with MD, especially when low-calorie and in combination with adequate physical activity. In addition, the composition of MD has been correlated with an excellent effect on reducing dyslipidemia. It also positively modulates the gut microbiota and immune system, significantly decreasing inflammatory mediators, a common ground for many obesity-related disorders.

***Summary*:**

People with obesity are at increased risk for a variety of medical disorders including hypertension, dyslipidemia, type 2 diabetes mellitus, and cardiovascular disease. Therefore, there is an inevitable need for measures to manage obesity and its related disorders. At this point, MD has been proposed as a valuable nutritional intervention. It is characterized by a high consumption of vegetables, fruit, nuts, cereals, whole grains, and extra virgin olive oil, as well as a moderate consumption of fish and poultry, and a limited intake of sweets, red meat, and dairy products. MD proves to be the healthiest dietary pattern available to tackle obesity and prevent several non-communicable diseases, including cardiovascular disease and type 2 diabetes.

## Introduction

The World Health Organization defines obesity as abnormal or excessive fat accumulation that portends increased risk to health status [1]. Despite this relatively simplistic definition, it is a chronic disease, a major public health problem that adversely effects all aspects of mental and physical health. Obesity has a growing worldwide prevalence irrespective of age, sex, race, or socioeconomic status [2]. Nearly a third of the world population is now suffering of overweight or obesity, which corresponds to one-fold increase since 1980 [3].

People with obesity are at increased risk for a variety of medical disorders including hypertension, dyslipidemia, type 2 diabetes mellitus, cardiovascular disease (CVD), and several cancers [4–6], all of which render obesity to be consistently associated with increased mortality [7, 8]. Therefore, there is inevitable need for measures to manage obesity and its related disorders. At this point, Mediterranean diet (MD) has been proposed to serve as a valuable nutritional intervention [9]. It is characterized by a high intake of vegetables, fruits, nuts, cereals, whole grains, and extra-virgin olive oil, as well as a moderate consumption of fish and poultry, and a limited intake of sweets, red meat, and dairy products [10]. Indeed, the adherence to MD dietary pattern is characterized by high intake of monounsaturated fat and fiber, and low in saturated fat with a balanced ratio of omega-6/omega-3 essential fatty acids [11]. The adherence to MD dietary pattern has been showed to be protective against the occurrence of several diseases, in particular obesity and CVD [12]. In particular, two previously conducted landmark randomized controlled trials (RCT) provided sobering evidence concerning the potential of MD for weight control and CVD prevention, which is not available for any other dietary pattern [13, 14]. Furthermore, Mediterranean dietary pattern compared to other diets has been reported as having proved to be the most effective in prevention of obesity and obesity-related diseases [15].

In this paper, we aimed to review the current knowledge regarding the effect of MD on obesity and obesity-related disorders.

## Mediterranean Diet and Body Composition

There is no single definition of what constitutes MD, but it generally consists of little amounts of red meat, low to moderate amounts of fish, poultry, and large quantities of fruit, vegetables, whole grains, and pulses with unrestricted olive oil as an important source of monounsaturated fatty acids (MUFA) [16]. This diet is generally considered to be relatively high in fat, and as such many health professionals may be reluctant to recommend it to individuals with overweight or obesity as high fat diets are perceived to promote weight gain. Contrary to that popular belief, epidemiological studies have described an inverse association of adherence to MD with Body Mass Index (BMI) and weight gain [17, 18]. Moreover, higher adherence to MD is associated with increased likelihood of weight loss maintenance [19]. However, most of those studies did not include assessments of physical activity or capture total energy intake, which could serve as significant confounders. The evidence from interventional studies on MD suggest that the weight effect depends more on energy content rather than macronutrient composition [20]. However, even when not energy restricted, this diet is not associated with weight gain. In the largest RCT on MD conducted to date, 7447 individuals were randomized to MD supplemented with olive oil, MD supplemented with nuts, or a low-fat diet and followed for a median of 4.8 years [21]. The fat content in the two MD arms accounted for 42% of daily energy. Calorie restriction was not required in either intervention arm and physical activity was not encouraged, despite high prevalence of overweight and obesity in the study population. At the end of follow-up, participants in each of the three groups had slightly reduced body weight and increased waist circumference (WC). In comparison with low-fat diet, neither arm of ad libitum MD demonstrated significant difference in body weight: −0.410 kg (95% CI −0.830 to 0.01; *p* = 0.056) for MD supplemented with olive oil and −0.016 kg (95% CI −0.453 to 0.421; *p* = 0.942) for MD supplemented with nuts. There was evidence that MD was associated with less gain of central adiposity as shown by the adjusted difference in WC after 5 years of −0.466 cm (95% CI −1.109 to 0.176; *p* = 0.154) in MD with olive oil and −0.923 cm (95% CI −1.604 to −0.241; *p* = 0.008) in the nut group, compared with low-fat diet group. In conclusion, high fat, unrestricted calories MD was associated with little weight changes and less central adiposity compared with low-fat diet long term [21]. A systematic meta-analysis of 16 RCTs (*n* = 3436) assessing MD interventions of duration between 4 weeks and 24 months concluded that consumption of MD is associated with a greater weight loss compared to control diets and that the weight loss is more significant when energy restriction and/or increased physical activity are recommended as part of the intervention [22]. Another systematic analysis assessed the effects of calorie-restricted MD on weight loss in individuals with overweight and obesity after 12 months or longer [23]. Five RCTs were included (*n* = 998). MD was somewhat superior in producing weight loss compared to low-fat diets (range of mean weight loss −4.1 to −10.1 kg vs. −2.9 to −5.0 kg) but (similar to) same as low-carbohydrate diet and the American Diabetes Association (ADA) diet. The effects of MD on BMI and WC were similar to that on body weight reduction [23]. The current recommendation for lifestyle management of subjects with type 2 diabetes and overweight or obesity is to achieve and sustain a weight loss of ≥ 5% a target which is often difficult to reach in clinical practice [24]. A meta-analysis incorporating 19 weight-loss intervention study groups (*n* = 2711) in type 2 diabetes demonstrated that energy restricted Mediterranean style diet combined with 175 min of physical activity weekly was one of only two interventions achieving the recommended 5% weight loss at 12 months [25]. This was associated with significant improvement in metabolic parameters [25]. Emerging evidence suggests that MD can reduce central adiposity and visceral fat, both of which have been associated with the risk of type 2 diabetes and CVD [26]. In cross-sectional studies, adherence to MD has been shown to be inversely associated with abdominal adiposity [17, 27, 28]. The beneficial effect of MD on reducing central adiposity and visceral fat could be related to its high content of polyunsaturated fatty acids (PUFA) and MUFA and low intake of saturated fatty acids (SFA) [11]. It has long been known that visceral adipose tissue comprises predominantly SFA, whereas subcutaneous fat has deposits of PUFA and MUFA [29]. In line with this hypothesis, a short cross-over study in patients with obesity (*n* = 11), individuals with insulin resistance demonstrated that an isocaloric MD rich in extra-virgin olive oil prevented central body fat accumulation compared with a low-fat diet without effect on body weight [30]. Reduction in visceral adipose tissue has been reported in two interventional trials of MD after 2 months [31, 32]. Contrary to these findings, a small RCT of ad libitum MD (*n* = 35) compared to a low-fat diet (*n* = 31) for 6 months demonstrated that the former was associated with reduced subcutaneous adipose tissue but not visceral adipose tissue or other body composition parameters in patients with overweight or obesity post coronary event [33]. However, the participants with more sustained adherence to MD had significantly lower WC (−2.81 cm, *p* = 0.01). No change in body weight, and a trend for reduction in total body fat, was observed despite the tendency for increased total energy intake in the MD group [33]. An intervention with calorie-restricted protein-enriched MD of 8 weeks’ duration has been shown to result in significant reduction in weight (−16.7%), visceral fat (−27.4%), and fat mass (−28.1%) with preservation of fat free mass (FFM) in men with obesity (*n* = 37) awaiting laparoscopic sleeve gastrectomy [32]. Another short-intervention study of 6 weeks’ duration demonstrated that hypocaloric MD was superior in reducing body fat mass and preserving FFM compared to high-protein diets in young, sedentary individuals [34••]. Preservation of FFM may be of particular importance in preserving short- and long-term benefits of weight loss given that FFM has been associated with decreased basal metabolic rate and the risk of developing sarcopenic obesity [35]. A meta-analysis of 50 studies including an overall population of nearly half a million subjects concluded that MD had beneficial effects on the risk of metabolic syndrome and its individual components, including WC (mean difference −0.42 cm; 95% CI −0.81 to −0.02) [36]. Importantly, larger effects were seen in trials conducted in Mediterranean countries, possibly due to better availability of the required food produce, although other confounders such as genetic or environmental factors may have played a role [36]. A systematic review looked specifically at the effects of MD on central obesity outcomes. The analysis of 18 interventional trials (7186 total subjects and 5168 subjects assigned to MD) concluded that MD could diminish abdominal adiposity as evidenced by reduction in WC, waist-hip ratio, or visceral fat [37]. The most consistent reductions were observed in WC and visceral fat, although only two studies reported the effects on the latter. The reduction in central adiposity, albeit not universal, was observed irrespective of whether energy restriction was recommended or not as part of the intervention. Interestingly, four of the five studies that did not observe improvement in measures of central adiposity were conducted in non-Mediterranean populations. It remains unclear if MD is more effective in reducing central adiposity compared to other dietary interventions given that MD showed superior effects only in three studies included in the analysis [37•]. In conclusion, MD is an effective tool in reducing body weight, particularly when energy restricted and in combination with increased exercise. Reassuringly, even when not energy-restricted it is not associated with weight gain in the short or long term.

MD has the potential to reduce abdominal adiposity, in particular metabolically detrimental visceral fat, independently of weight loss, and can be recommended as a healthy diet choice to individuals with obesity and overweight, particularly at risk of cardiovascular and metabolic disease. MD may be more effective in Southern European populations due to better availability of specific food produce, cultural and other factors.

## Mediterranean Diet and Type 2 Diabetes

The International Diabetes Federation (IDF) estimated just over 20 years ago that 151 million adults were affected by T2D worldwide [38]. This has increased to 463 million in 2019, suggesting a tripling of the global burden over this period [38].

**Fig. 1:**
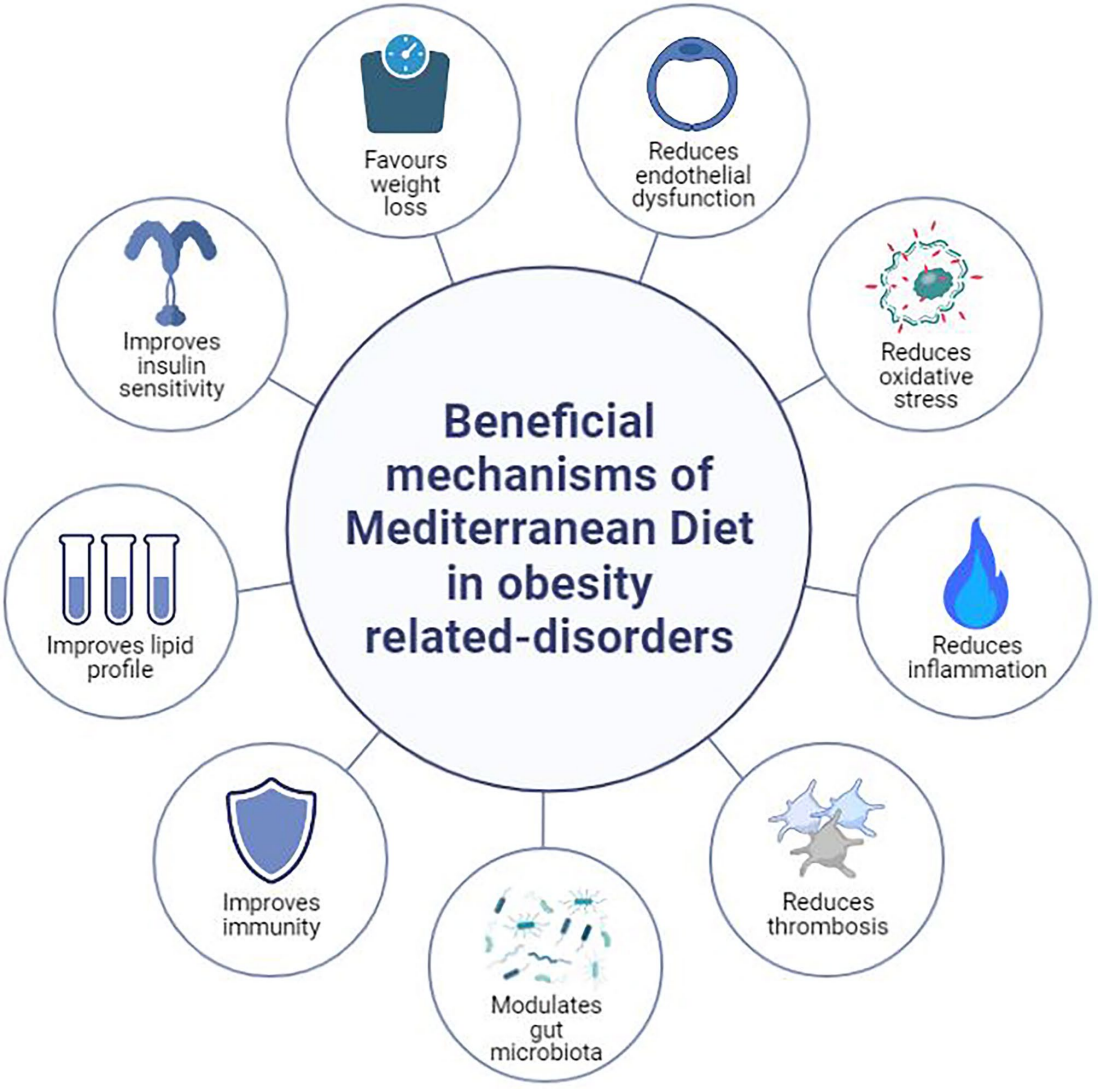
The beneficial mechanisms of Mediterranean diet in obesity-related disorders

Lifestyle measures remain the cornerstone for type 2 diabetes treatment as recommended by several scientific societies, including the ADA and the European Association for the Study of Diabetes (EASD) [39, 40]. In the latest (2021) ADA guidelines, recommendations for medical nutrition therapy emphasize the implementation of a Mediterranean-style eating pattern to improve both glucose and lipid metabolism, thus minimizing individual’s cardiovascular risk [39]. Such an eating pattern is characterized by reduced consumption of saturated and trans-fat, as well as an increased intake of dietary PUFA n-3, viscous fiber, and plant sterols/stanols. The plant-based components of MD (e.g., vegetables, fruits, whole grains, and nuts) contain polyphenols that have been shown to reduce insulin resistance and improve cardiometabolic risk factors [41]. Olive oil and low-to-moderate alcohol intake (especially red wine) also contribute to the benefits of MD via their polyphenol content [42, 43]. Overall, potential mechanisms underlying the beneficial effects of MD include improvements in oxidative stress, inflammation, thrombosis, insulin sensitivity, lipid profile, endothelial dysfunction, and gut microbiota [44–46] (Fig. 1).

## Mediterranean Diet and Type 2 Diabetes Prevention

Type 2 diabetes is considered one of the major obesity-related comorbidities. The core pathophysiologic defect which is at the base of obesity is insulin resistance in muscle and liver, predicting the onset of type 2 diabetes in susceptible subjects [47]. Although current dietary recommendations focused on weight loss and overall dietary quality, to date, in subjects with obesity, there is no consensus on the optimal macronutrient composition of the diet for long-term management of type 2 diabetes [48]. Previous meta-analyses showed that adherence to MD could prevent type 2 diabetes development by 19–23% [49, 50]. In a more recent cohort study (*n* = 25,317 female participants from the Women’s Health Study), a higher MD intake was related to a 30% relative risk decrease in type 2 diabetes incidence during a 20-year follow-up [51]. Furthermore, an inverse association between adherence to MD and the prevalence of metabolic syndrome and prediabetes has been reported [52, 53].

## Mediterranean Diet and Cardiometabolic Risk Factors

In patients with type 2 diabetes, MD can beneficially affect glycemic control and cardiovascular risk [54, 55]. In this context, a cross-sectional study among 500 patients with type 2 diabetes investigated the impact of MD on glycated hemoglobin (HbA1c). Mean HbA1c was 8.57 (±standard deviation, SD 1.94), 7.63 (±1.32), and 6.47 (±0.7) % in patients with low, moderate, and high adherence to MD, respectively [56]. In another randomized trial (*n* = 215 newly diagnosed patients with type 2 diabetes), HbA1c was significantly reduced by 1.2 and 0.9% at year 1 and 4, respectively, in patients on MD [57]. Fasting plasma glucose, serum insulin levels, and Homeostasis Model Assessment of Insulin Resistance (HOMA-IR) were also significantly decreased. As a consequence, significantly less patients needed antidiabetic drug therapy both at year 1 and year 4 (hazard ratio, HR 0.70, 95% CI 0.59 to 0.90) [57]. Similar results were reported in a systematic review of 20 RCTs (> 6 months duration, *n* = 2223 patients with type 2 diabetes) showing greater decreases in body weight and HbA1c levels, as well as delayed requirement for antidiabetic drugs in patients with type 2 diabetes following a MD compared with those on other low-fat or low-carbohydrate diets [58].

Apart from improvements in glucose metabolism and body weight, MD can beneficially affect other cardiovascular risk factors, including lipids as triglycerides (TG), low-density lipoprotein cholesterol (LDL-C) and high-density lipoprotein cholesterol (HDL-C), and blood pressure (BP), in patients with type 2 diabetes [59]. For example, among 2568 patients with type 2 diabetes, those with a high MD score had significantly lower LDL-C (101.5 ± 31.2 vs. 105.1 ± 31.9 mg/dL), TG (146.7 ± 71.0 vs. 156.2 ± 78.6 mg/dL), systolic BP (133.3 ± 23.7 vs. 135.3 ± 14.9 mmHg), and diastolic BP (78.6 ± 8.5 vs. 80.7 ± 8.7 mmHg), as well as higher HDL-C (46.8 ± 12.4 vs. 45.3 ± 11.6 mg/dL) than those with a low MD score [60]. Such findings were confirmed in a network meta-analysis (10 RCTs, *n* = 921 patients with type 2 diabetes) reporting that MD was superior to a low-fat diet in reducing HbA1c (mean difference −0.45%; 95% CI −0.55 to −0.34), fasting plasma glucose (mean difference −1.24 mmol/L; 95% CI −1.57 to −0.91), weight (mean difference −1.18 kg; 95% CI −1.99 to −0.37), WC (mean difference −0.73 cm; 95% CI −1.26 to −0.19), and TG (mean difference −0.21 mmol/L; 95% CI −0.27 to −0.16), as well as in increasing HDL-C (mean difference 0.07 mmol/L; 95% CI 0.04 to 0.11) [61]. Similarly, in another meta-analysis (9 RCTs, *n* = 1178 type 2 diabetes patients), MD led to greater decreases in HbA1c (mean difference −0.30%; 95% CI −0.46 to −0.14), fasting plasma glucose (mean difference −0.72 mmol/L; 95% CI −1.24 to −0.21), fasting insulin (M mean difference −0.55 μU/mL; 95% CI −0.81 to −0.29), weight (mean difference −0.29 kg; 95% CI −0.55 to −0.04), BMI (mean difference −0.29 kg/m^2^; 95% CI −0.46 to −0.12), TG (mean difference −0.29 mmol/L; 95% CI −0.47 to −0.10), systolic BP (mean difference −1.45 mmHg; 95% CI −1.97 to −0.94), and diastolic BP (mean difference −1.41 mmHg; 95% CI −1.84 to −0.97), as well as greater increases in HDL-C (mean difference 0.06 mmol/L; 95% CI 0.02 to 0.10) compared with control diets [62]. These cardiometabolic effects of MD in patients with type 2 diabetes were also summarized in a previous systematic review [63].

## Mediterranean Diet and Diabetic Microvascular Complications

Low adherence to MD was also linked to impaired renal function and health-related quality of life in patients with type 2 diabetes and chronic kidney disease [64, 65]. Among women with type 2 diabetes, moderate and high MD scores were related to significantly reduced rates of diabetic nephropathy by 62% (OR 0.38; 95% CI 0.20 to 0.73) and 86% (OR 0.14; 95% CI 0.06 to 0.33), respectively, compared with a low MD score [66]. Of note, increases in MD score by 1-point were associated with 10% lower risk of CKD (mean follow-up 20.6 ± 7.0 years) as shown in a meta-analysis (13 studies, *n* = 27,618 individuals) [67]. Furthermore, implementation of MD was associated with decreased rate of incident CKD during mean follow-up of 24 years among 12,155 participants (aged 45–64 years) from the Atherosclerosis Risk in Communities Study [68]. Even when CKD has been developed, MD can exert nephroprotection. For example, in a cross-sectional analysis of the German Chronic Kidney Disease Study (*n* = 2813 patients with CKD), a high MD score correlated with higher estimated glomerular filtration rate (eGFR) (β-coefficient 0.932, *p* = 0.007) [64]. Overall, MD has been shown to prevent CKD, as well as decrease renal function decline and improve survival in patients with CKD [69].

Increased adherence to MD has been associated with lower risk of developing diabetic retinopathy (HR 0.34; 95% CI 0.13 to 0.89; *p* = 0.001 for trend) among 3614 patients with type 2 diabetes (aged 55–80 years) from the PREvención con DIeta MEDiterránea (PREDIMED) study [70]. Similar results have been reported in other studies [71–73].

Few evidence supports a link between adherence to MD and protection against diabetic neuropathy development [72], but further research is needed to elucidate such associations. Similarly, there is data showing that MD may preserve cognitive function and prevent dementia [74, 75], even in patients with type 2 diabetes [76].

## Mediterranean Diet and Diabetic Macrovascular Complications

A previous umbrella review of meta-analyses found that high adherence to MD was associated with a reduced risk of overall mortality, CVD, coronary heart disease (CHD), myocardial infarction (MI), overall cancer incidence, type 2 diabetes, and neurodegenerative diseases [77]. Furthermore, MD was reported to protect against worse outcomes (heart failure hospitalization, unstable angina, stroke, recurrent MI, all cause or cardia death) up to 46 months following an MI [78]. Similarly, data from the GISSI-Prevenzione clinical trial showed that MD significantly decreased all-cause death in 11,323 patients with MI [79]. Furthermore, among 23,232 participants of the European Prospective Investigations into Cancer and Nutrition (EPIC) study, followed up for 17 years, stroke risk was significantly reduced with a greater adherence to MD (HR 0.78; 95% CI 0.65 to 0.93) [80]. Low adherence to MD was also related to a higher incident of stroke compared with moderate (HR 1.32; 95% CI 1.05 to 1.66) and high adherence (HR 1.28; 95% CI 1.00 to 1.63) among 30,239 participants of the REasons for Geographic And Racial Differences in Stroke (REGARDS) study, followed up for 6.5 years [81]. MD was shown to protect against peripheral artery disease (PAD) development in the PREDIMED study (*n* = 7435 participants, median follow-up: 4.8 years) [82].

In patients with type 2 diabetes, following MD led to significant reductions in CVD incidence (RR 0.62; 95% CI 0.5 to 0.78) [83]. A recent meta-analysis of 38 cohort studies and 3 RCTs (including patients with type 2 diabetes) found that adherence to MD was associated with significantly lower incidences of CHD (RR 0.73; 95% CI 0.62 to 0.86), MI (RR 0.73; 95% CI 0.61 to 0.88), and stroke (RR 0.80; 95% CI 0.71 to 0.90), as well as of CHD mortality (RR 0.83; 95% CI 0.75 to 0.92), stroke mortality (RR 0.87; 95% CI 0.80 to 0.96), and total CVD death (RR 0.79; 95% CI 0.77 to 0.82) [84]. A higher MD score was also linked to a significantly lower risk (by 66%) of PAD incidence (OR 0.44; 95% CI 0.24 to 0.83) in patients with type 2 diabetes (*n* = 944) [85]. Similarly, increased intake of fish and shellfish was reported to marginally decrease PAD risk (HR per additional gram/week 0.99; 95% CI 0.99 to 1.00, *p* = 0.051) in 1112 patients with type 2 diabetes followed for a median of 19.7 years in the Malmö Diet and Cancer study [86]. Nevertheless, further clinical data is needed in patients with type 2 diabetes to establish the effects of MD on cardiovascular morbidity and mortality.

In sum, in patients with type 2 diabetes, MD may minimize the risk of diabetic micro and macrovascular complications, although further evidence is required. Therefore, implementation of MD is recommended for both prevention and treatment of prediabetes and type 2 diabetes [55, 87, 88].

## Mediterranean Diet and Non-alcoholic Fatty Liver Disease

Non-alcoholic fatty liver disease (NAFLD) has been associated with type 2 diabetes and further increase of cardiovascular risk [89–92]. NAFLD is regarded as the hepatic manifestation of metabolic syndrome [93]. Currently, the global NAFLD prevalence is reported to be 25%, being highest in the Middle East (32%) and South America (31%) followed by Asia (27%), the USA (24%), Europe (23%), and Africa with the lowest prevalence (14%) [94].

MD has been proposed as an effective nutrition therapy for patients with type 2 diabetes and/or NAFLD, representing the first-line treatment for these metabolic diseases [93, 95•, 96]. In this context, MD was shown to improve biochemical and histological features of NAFLD [97, 98]. Therefore, implementation of MD may protect liver structure and function in patients with type 2 diabetes. Of interest, in patients with of NAFLD, it has been reported the enlargement of spleen which is the central organ in regulating the inflammation-related immune response depicting the so called liver-spleen axis [99]. Healthy dietary patterns, including MD, have been reported to improve immune and inflammatory responses by both reducing NAFLD and improving spleen function [100].

**Fig. 2:**
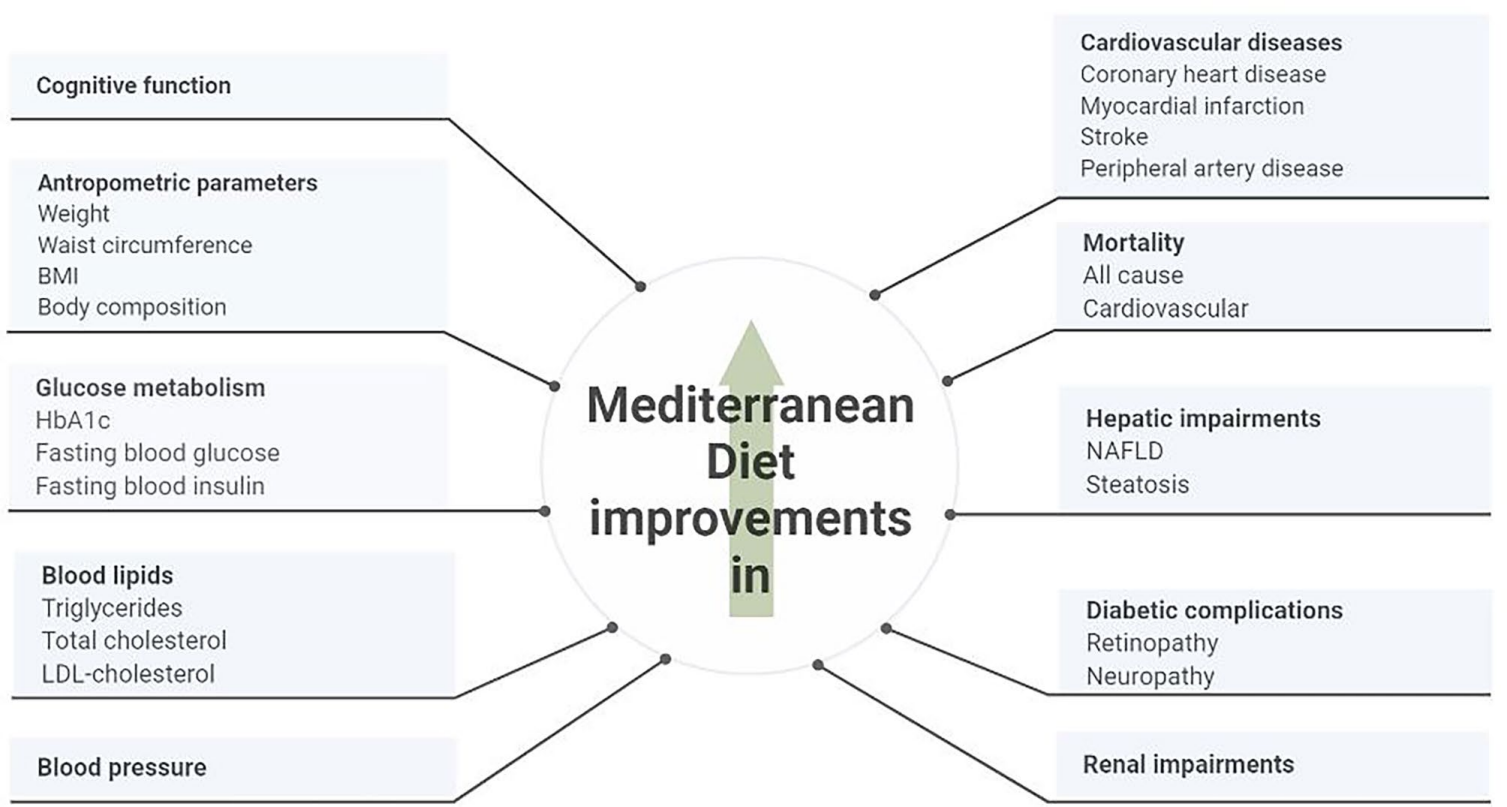
The beneficial effects of adherence to MD on cardiometabolic factors and diabetic complications in patients with type 2 diabetes

Overall, adherence to MD exerts several health benefits by improving cardiometabolic risk factors, including glucose and lipid metabolism, obesity indexes and NAFLD (Fig. 2).

## Mediterranean Diet and Dyslipidemia

Dyslipidemia is a primary cause of the atherosclerotic cardiovascular disease (ASCVD) [101]. In particular, the most atherogenic form has been associated with type 2 diabetes and insulin resistance conditions [102]. Dyslipidemia is characterized by elevated serum levels of LDL-C and TG and low levels of HDL-C [103]. In 2008, according to the WHO Global Health Observatory, the prevalence of a plasma total cholesterol level ≥ 190 mg/dl was highest in Europe (54% for both sexes), followed by North and South America (48% for both sexes), while Africa and South-East Asia had the lowest prevalence (22.6% and 29.0%, respectively) [104]. Between 1980 and 2018, globally, little or no change in total and non-HDL plasma cholesterol was observed, but several regions experienced significant changes in some lipid parameters: high-income countries, which had the highest plasma cholesterol levels in 1980, experienced a substantial reduction in plasma cholesterol levels, while low- and middle-income countries experienced large increases in both plasma cholesterol and plasma triglycerides [105].

In recent years, several studies have investigated the role of diet and dyslipidemia. MD is a dietary pattern recommended for cardiovascular prevention and has been promoted by the European Society of Cardiology/European Atherosclerosis Society (ESC/EAS) 2019 guidelines for the management of dyslipidemia in addition to other lifestyle changes (Table 1) [103]. Ancel Keys designed MD almost five decades ago, and it has been recognized as one of the healthiest dietary patterns [16]. In addition, it has been negatively related to various chronic diseases, such as CVD [106], cancer [107], obesity [108], type 2 diabetes [88], and other metabolic conditions [109].

**Table 1:** Treatment targets/goals 2019 European Society of Cardiology/European Atherosclerosis Society (ESC/EAS) guidelines [103]

**Treatment**	**Recommendation**
*Smoking*	No use in any form of tobacco
*Diet*	Diet low in saturated fat with whole grain products, vegetables, fruit, and fish
*Physical activity*	30–60 min moderately vigorous physical activity most days
*Bodyweight*	BMI 20–25 kg/m^2^, and WC < 94 cm and < 80 cm (women)
*Blood pressure*	< 140/90 mmHg

*BMI* body mass index, *WC* waist circumference

Specifically for patients that suffer from hyperlipidemia, MD advocates for low intake of SFA, less than < 7% in patients with hypercholesterolemia, and high consumption of PUFA and micronutrients, including dietary vitamins and minerals, that rises the plasma antioxidant capacity [110]. Also, MD favors the restriction of milk and dairy product consumption and limited intake of meat and meat-derived products [16]. Moreover, high plant-based food intake, such as whole grains, vegetables, and fruits, is highly advisable. MD suggests the consumption of seafood, regular consumption of olive oil, and increased physical activity. Finally, MD recommends reducing simple sugar intake and eliminating alcohol. This dietary pattern is high on food groups such as fruits and vegetables, fibers, olive oil, fish, and red wine, that are rich sources of several bioactive compounds, including antioxidants like carotenoids, flavonoids, resveratrol, and other polyphenolic compounds [16].

Studies have proven that consumption of foods causes modifications in the gut microbiota leading to an increase of beneficial bacteria such as *Lactobacillus, Bifidobacterium*, and *Prevotella*, and a reduction of harmful bacteria like *Clostridium* [111]. This effect is positive for prevention and treatment of chronic diseases like obesity [112], dyslipidemia [113], and inflammation [114]. MD is also considered a high fiber intake pattern, which can act on gut microbiota by modulating its composition and the production of metabolites that regulate immune function [115]. MD leads to an increase in the number of intestinal bacterial species responsible for producing short-chain fatty acids (SCFAs) such as acetate, propionate, and butyrate, essential for proper functioning in preventing metabolic diseases [115]. Also, high consumption of PUFA n-3 from fish and vegetable sources and an adequate PUFA n-6/ PUFA n-3 ratio from MD [116] promotes a better metabolic profile compared to other dietary patterns that are high on PUFA n-6 and favors a higher production of proinflammatory substances and that increases the risk of chronic diseases like ASCVD [117]. Dietary fiber has become a key mediator of the communication between the brain and the gut [118]. SCFAs exert their beneficial effects directly by contributing to modulation of host health through a range of tissue-specific mechanisms related to gut barrier function, glucose homeostasis, immunomodulation, and appetite regulation [118]. Evidence on this effect has emerged from the modulation of gut microbial composition through administration of prebiotics, defined as a non-digestible food ingredient that stimulates the growth and/or activity of one or a limited number of beneficial bacteria in the colon [119].

In addition to the gut microbiota modulation effects, the beneficial impact of MD could also be due to immune system modulation [100, 120]. In an intervention study conducted by Llorente-Cortés et al. in 2010 on a population with a high risk of ASCVD (*n* = 49), it was found that after 3 months, subjects who followed MD integrated with virgin olive oil or nuts showed not only a reduction in interleukin-6 and soluble intercellular adhesion molecule-1, significant inflammation mediators in the adhesion of leukocytes to the endothelial surface, but also a reduction in activation of biomarkers related to the atherosclerotic process [120]. There was a reduction of the proinflammatory ligand CD40 and the adhesion molecule CD49d on T lymphocytes and monocytes after both MD [120].

Different researchers have studied the relationship between SFA consumption, ASCVD risk and increased LDL-C levels [121], while recent clinical studies support the fact that SFA do not increase the risk of ASCVD. The relationship between SFA intake and cardiovascular mortality has been discussed previously, and there was no higher risk of ASCVD events in individuals with high consumption of SFA than those with low consumption [121]. It has been concluded that ASCVD risk may be more influenced by the dietary source of SFA, mainly giving dairy and meat products more focus [121]. Therefore, a higher intake of SFA from meat products is related to the development of ASCVD, and that lower ASCVD risk is linked to a higher intake of SFA from dairy sources. However, the literature is still controversial regarding the relationship between meat and dairy products intake and the effect on lipid profile [88, 121, 122]. The Prospective Urban and Rural Epidemiology study was a large study that helped understand the connection between macronutrient intake and mortality, concluding that SFA intake does not influence mortality rate [123]. At the same time, high consumption of carbohydrates has been associated with a higher mortality risk for CVD [123].

Other studies have investigated the relationship between SFA intake and cardiovascular mortality and did not observe an increased risk of ASCVD events in subjects with high consumption of SFA compared with those with low consumption [124–126]. Consequently, ASCVD risk may be influenced by the dietary source of SFA, mainly represented by dairy and meat products. Meat consumption is a dietary risk factor for atherogenic dyslipidemia [126]. de Oliveira et al. in the Multi-Ethnic Study of Atherosclerosis (*n* = 5209) reported that a higher intake of SFA from meat products is related to the development of ASCVD; on the other hand, a lower ASCVD risk has been correlated to a higher intake of SFA from dairy products [125]. However, studies are controversial regarding the relationship between meat and dairy products intake and the effect on lipid profile [125, 126].

In 2021, Formisano et al. evaluated the influence of different eating habits on the lipid profile of 106 patients suffering from different types of dyslipidemia [126]. They concluded that a high intake of dairy products was associated with hyperlipidemia (higher levels of total cholesterol and HDL-C), while a diet with an excessive amount of meat products caused a form of dyslipidemia (higher total cholesterol and TG levels and lower HDL-C levels) [126]. Therefore, dietary recommendations should specify between SFA other than suggesting general reduction in SFA intake.

Adherence is primordial for treatment of chronic diseases [88, 122, 127]. Some reports examine the adherence to MD and have shown that individuals with obesity and with low adherence to MD presented worse anthropometric measurements and metabolic profile compared with subjects who were good sleepers and with an average adherence, independently of age and gender [88, 122, 127]. Also, the effect on gut microbiota includes the maintenance of presence of *Prevotella* bacteria and other *Firmicutes* according to the degree of adherence to MD [127–129]. On the contrary, low adherence to MD has been associated with high levels of urinary trimethylamine N-oxide, which is related to increased cardiovascular risk [130, 131]. Of interest, through inflammatory processes, TMAO would have a potential role in different chronic non-communicable diseases, including obesity [132], CVD [133], type 2 diabetes [134], NAFLD [134], and inflammatory diseases [135]. Also, obesity is associated with reduced spontaneous and stimulated growth hormone secretion and basal insulin-like growth factor I levels [136] which has been associated with increased risk of ASCVD [137]. The degree of adherence to MD and protein grams intake was one of the most predictive factors of growth hormone status in obesity, showing an association between adherence to MD and the clinical alterations of cardiometabolic status [136].

Moreover, another critical nutrient that MD considers is vitamin D, and the evidence suggests that vitamin D deficiency may represent a significant risk factor [138–140]. There is a close relationship between vitamin D and the cardiovascular system by vitamin D receptors in vital tissues like endothelium, smooth muscle, and myocardium [138–140]. Therefore, low vitamin D status has been associated with increased BP [139, 140], dyslipidemia [139, 140], impaired insulin metabolism [138], sleep disturbances [141, 142], thus increasing the risk of cardiovascular atherosclerosis [139, 140]. It has been studied that hypovitaminosis D might increase the cardiovascular risk in hypopituitarism patients, and it is a powerful predictor of prevalence of dyslipidemia and hypertension in individuals [138, 140]. Hypovitaminosis D is commonly reported in patients with obesity due to several mechanisms [143, 144]. Of interest, very recently in a cross-sectional, observational study, it was reported that high adherence to MD was associated with low BMI in 617 individuals, probably through the antioxidant and anti-inflammatory effects synergistically exerted by either high vitamin D levels or high adherence to MD on body weight [145••].

## Mediterranean Diet and Cancer

To date, the important role of prevention in several cancer settings is well known, and diet has a good place among these prevention strategies. A meta-analysis including 2,130,753 participants concluded that greater adherence to MD was associated with a significantly lower risk of cancer mortality (RR 0.86; 95% CI 0.81 to 0.91), colorectal cancer (RR 0.82; 95% CI 0.75 to 0.88), breast cancer (RR 0.92; 95% CI 0.87 to 0.96), gastric cancer (RR 0.72; 95% CI 0.60 to 0.86), liver cancer (RR 0.58; 95% CI 0.46 to 0.73), head and neck cancer (RR 0.49; 95% CI 0.37 to 0.66), and prostate cancer (RR 0.96; 95% CI 0.92 to 1.00) [146]. In addition, data on the benefits of MD against incident cancers were reported in the EPIC trial (*n* = 9669 incident cancers in men and 21,062 in women) [147]. Evidence of protection was strongest for colorectal, gastric, and breast cancers, especially after exclusion of alcohol from the score [147].

Breast cancer has increased by more than 20% worldwide since 2008 and is the leading cause of cancer in women [148]. Data from the PREDIMED study showed that after a mean follow-up of 4.8 years, the observed rates of breast cancer were lower in the intervention groups with EVOO or with nuts than in the control group with a low-fat diet. After multivariate adjustment, the group supplementing MD with EVOO had a significantly lower risk of developing breast cancer than the control group; for every 5% additional calories from EVOO, the risk was 28% lower (95% CI, 0.57 to 0.90) [148]. A case–control study of 2396 women aged 25–74 years found that MD was associated with a 35% reduction in breast cancer risk [149]. The Four Corners Breast Cancer Study showed that Hispanic (*n* = 757 cases and 867 controls) and non-Hispanic (*n* = 1524 cases and 1598 controls) women who adopted MD had a lower risk of breast cancer [150]. Two prospective studies of 91,779 American women [151] and 65,374 French women [152] confirmed a protective association between adherence to MD and breast cancer incidence. The protective effect of MD on breast cancer risk was associated with a reduction in circulating estrogen levels and increased intake of carotenoids, which are known antioxidants that reduce oxidative stress. The protective associations were greater in women with negative progesterone and estrogen receptor [152].

A lower risk of colon cancer has been associated with dietary patterns that are higher in vegetables, legumes, fruits, whole grains, fish, lean meats, low-fat dairy products, moderate alcohol consumption, and lower consumption of red and/or processed meats, sugar-sweetened beverages, and saturated fats [153]. In contrast, diets containing greater amounts of red/processed meat, sugars (i.e., desserts, sugar-sweetened beverages, and sweets), potatoes, and chips are associated with an increased risk of colorectal cancer [153]. Data from the Italian EPIC study involving 42,275 participants aged 25–70 years who did not have cancer at baseline found that increased adherence to MD was associated with an 8–11% lower risk of colorectal cancer in men and women [154]. The protective effect was observed mainly for distal colon and rectal cancer, whereas it was lower for proximal colon cancer [154].

In conclusion, all the above results suggest that adherence to MD may contribute to the reduction of various cancers and also of overall cancer-related mortality. Nevertheless, further research is needed to determine which foods and nutrients are most effective for this outcome (Table 2).

**Table 2:** Meta-analyses of studies regarding the effects of Mediterranean diet on obesity-associated disorders

**Source**	**No. and type of studies**	**Subjects**	**Aim**	**Main findings**
Esposito et al. [22]	16 RCTs	3436 subjects	To evaluate the effect of MD on body weight	MD had a significant effect on weight (95% CI −2.86 to −0.64) and BMI (95% CI −0.93 to −0.21). The effect of MD on body weight was greater in association with energy restriction (mean difference, −3.88 kg, 95% CI −6.54 to −1.21 kg), increased physical activity (−4.01 kg, 95% CI −5.79 to −2.23 kg), and follow-up longer than 6 months (−2.69 kg, 95% CI −3.99 to −1.38 kg)
Franz et al. [25]	11 RCTs	6754 adults with overweight or obesity and T2DM	To evaluate the outcomes on HbA1c, lipid (total cholesterol, LDL-C, HDL-C, and TG) and BP (systolic and diastolic) from lifestyle weight-loss interventions resulting in weight losses greater than or less than 5% at 12 months To evaluate the weight and metabolic outcomes from differing amounts of macronutrients in weight-loss interventions	2 study groups reported a weight loss of ≥ 5%: a Mediterranean-style diet implemented in newly diagnosed adults with T2DM, and an intensive lifestyle intervention implemented in the Look AHEAD trial. Both included regular physical activity and frequent contact with health professionals and reported significant beneficial effects on HbA1c, lipids, and blood pressure
Kastorini et al. [36]	50 RCTs (35 clinical trials, 2 prospective and 13 cross-sectional)	534,906 subjects	To meta-analyze epidemiological studies and clinical trials that have assessed the effect of MD on metabolic syndrome as well as its components	Adherence to MD was associated with reduced risk of metabolic syndrome (log HR −0.69; 95% CI −1.24 to −1.16). Results from clinical studies revealed the protective role of MD on components of metabolic syndrome, like WC (mean difference −0.42 cm; 95% CI −0.82 to −0.02), HDL-C (mean difference 1.17 mg/dl; 95% CI 0.38 to 1.96), TG (mean difference −6.14 mg/dl; 95% CI −10.35 to −1.93), systolic (mean difference −2.35 mm Hg; 95% CI −3.51 to −1.18) and diastolic BP (mean difference −1.58 mm Hg; 95% CI −2.02 to −1.13), and glucose (mean difference −3.89 mg/dl; 95% CI −5.84 to −1.95), whereas results from epidemiological studies also confirmed those of clinical trials
Koloverou et al. [49]	10 prospective studies (1 clinical trial, 9 prospective and 7 cross-sectional)	136,846 subjects	To meta-analyze prospective studies that have evaluated the effect of MD on the development of T2DM	Higher adherence to MD was associated with 23% reduced risk of developing T2DM (combined RR for upper vs lowest available centile: 0.77; 95% CI 0.66 to 0.89). Subgroup analyses based on region, health status of participants and number of confounders controlling for, showed similar results
Schwingshackl et al. [50]	1 RCT and 8 prospective cohort studies	122,810 subjects	To meta-analyze the effects of MD adherence on the risk of T2DM	For highest vs lowest adherence to MD score, the pooled RR for T2DM was 0.81 (95% CI 0.73 to 0.90). Sensitivity analysis including only long-term studies confirmed the results of the primary analysis (pooled RR 0.75; 95% CI 0.68 to 0.83)
Pan et al. [61]	10 RCTs	921 subjects with T2DM	To comprehensively compare the differences between major dietary patterns in improving glycemic control, cardiovascular risk, and weight loss for patients with T2DM	Compared to low-fat diet, MD showed beneficial effects in glycemic control (HbA1c 95% CI –0.55 to –0.34; fasting plasma glucose 95% CI –1.57 to –0.91; weight loss 95% CI –1.99 to –0.37; WC 95% CI –1.26 to –0.19), and cardiovascular risk factors (HDL-C 95% CI 0.04 to 0.11; total cholesterol 95% CI –0.26 to –0.08; TG 95% CI –0.27 to –0.16)
Huo et al. [62]	9 RCTs	1178 subjects with T2DM	To explore the effects of MD on glycemic control, weight loss and cardiovascular risk factors in T2DM patients	Compared with control diets, MD led to greater reductions in HbA1c (mean difference, −0.30; 95% CI −0.46 to −0.14), fasting plasma glucose (−0.72 mmol/l; 95% CI −1.24 to −0.21), fasting insulin (−0.55 μU/ml; 95% CI −0.81 to −0.29), BMI (−0.29 kg/m^2^; 95% CI −0.46 to −0.12) and body weight (−0.29 kg; 95% CI −0.55 to −0.04). Likewise, concentrations of total cholesterol and TG were decreased (−0.14 mmol/l; 95% CI −0.19 to −0.09 and −0.29 mmol/l; 95% CI −0.47 to −0.10, respectively), and HDL-C was increased (0.06 mmol/l; 95% CI 0.02 to 0.10). In addition, MD was associated with a decline of 1.45 mm Hg (95% CI −1.97 to −0.94) for systolic and 1.41 mm Hg (95% CI −1.84 to −0.97) for diastolic BP
Hansrivijit et al. [67]	4 prospective studies	8467 subjects ≥ 18 years of age without CKD	To assess the association between MD adherence and CKD prevention	With the mean follow-up duration of 20.6 ± 7.0 years, the pooled OR for CKD was 0.901 (95% CI 0.868 to 0.935) for each 1-point increment of MD scale. The incidence of CKD was 0.026 events per person-year (95% CI 0.008 to 0.045)
Becerra-Tomás et al. [84]	3 RCTs and 38 prospective cohort studies	Adults with type 1 diabetes or T2DM	To evaluate the effect of MD on the prevention of CVD incidence and mortality	Meta-analyses of RCTs revealed a beneficial effect of MD on total CVD (RR: 0.62; 95% CI 0.50 to 0.78) and total myocardial infarction (RR: 0.65; 95% CI 0.49 to 0.88) incidence Meta-analyses of prospective cohort studies, which compared the highest vs lowest categories of MD adherence, revealed an inverse association with total CVD mortality (RR 0.79; 95% CI 0.77 to 0.82), CHD incidence (RR 0.73; 95% CI 0.62 to 0.86), CHD mortality (RR 0.83; 95% CI 0.75 to 0.92), stroke incidence (RR 0.80; 95% CI 0.71 to 0.90), stroke mortality (RR 0.87; 95% C: 0.80 to 0.96) and myocardial infarction incidence (RR 0.73; 95% CI 0.61 to 0.88)
Schwingshackl et al. [146]	2 RCTs, 51 cohort studies and 30 case–control studies	2,130,753 subjects	To evaluate the effects of adherence to MD on risk of overall cancer mortality, risk of different types of cancer, and cancer mortality and recurrence risk in cancer survivors	Greater adherence to MD was associated with a significantly lower risk of cancer mortality (RR 0.86; 95% CI 0.81 to 0.91), colorectal cancer (RR 0.82; 95% CI 0.75 to 0.88), breast cancer (RR 0.92; 95% CI 0.87 to 0.96), gastric cancer (RR 0.72; 95% CI 0.60 to 0.86), liver cancer (RR 0.58; 95% CI 0.46 to 0.73), head and neck cancer (RR 0.49; 95% CI 0.37 to 0.66), and prostate cancer (RR 0.96; 95% CI 0.92 to 1.00)

*RCT* randomized controlled trial, *MD* Mediterranean diet, *CI* confidence interval, *BMI* body mass index, *T2DM* type 2 diabetes mellitus, *LDL-C* low-density lipoprotein cholesterol, *HDL-C* high-density lipoprotein cholesterol, *BP* blood pressure, *AHEAD* Action for Health in Diabetes, *HR* hazard ratio, *WC* waist circumferences, *RR* risk ratio, *CKD* chronic kidney disease, *CVD* cardiovascular disease, *CHD* coronary heart disease

## Conclusion

The obesity pandemic is associated with high risk of morbidity and mortality from different non-communicable diseases. Of interest, the negative effects of obesity are reversed in part with substantial weight loss. The composition of MD has been related to an excellent effect on reducing dyslipidemia. Additionally, it positively modulates the gut microbiota and immune system, significantly decreasing inflammation mediators, common ground for many obesity-related disorders. MD is the healthiest dietary pattern available to prevent several non-communicable diseases, including cardiovascular disease and type 2 diabetes.
